# Nitrogen and Sulfur Fertilization Modulates the Yield, Essential Oil and Quality Traits of Wild Marigold (*Tagetes minuta* L.) in the Western Himalaya

**DOI:** 10.3389/fpls.2020.631154

**Published:** 2021-01-18

**Authors:** Swati Walia, Rakesh Kumar

**Affiliations:** ^1^Academy of Scientific and Innovative Research, Ghaziabad, India; ^2^Agrotechnology Division, CSIR-Institute of Himalayan Bioresource Technology, Council of Scientific and Industrial Research, Palampur, India

**Keywords:** wild marigold, yield, nitrogen, sulfur, oil glands, quality, *Z-β-*ocimene, NUE

## Abstract

Fertilization plays an irreplaceable role in raising crop yields; however, there are issues with unnecessary and blind use of chemical fertilizers, which raise the risk of contamination in the atmosphere. It is hypothesized that fertilization of nitrogen (N) and sulfur (S) will together improve the essential oil (EO) yield and composition of *Tagetes minuta* L. Thus, 2 years field experiment were carried out to evaluate the outcomes of N (0, 60, 90, and 120 kg ha^–1^) and S levels (0, 20, 40, and 60 kg ha^–1^) on *T. minuta* during 2018 and 2019. The growth, biomass, EO content and composition were influenced (*P* = 0.05) by N and S fertilization. N at 120 kg ha^–1^ and S at 60 kg ha^–1^ registered higher biomass (183.89 and 178.90 q ha^–1^, respectively) and EO yield (102.09 and 88.60 kg ha^–1^, respectively), than control. Stomatal density reduced significantly with increase of N and S levels, however, density of oil glands substantially increased with S at 40 and 60 kg ha^–1^. The major component of EO (*Z*-β-ocimene) significantly increased with 120 kg N ha^–1^ (42.59%) and 60 kg S ha^–1^ (42.35%), respectively. Available nutrients in soil and plant tissues substantially increased with N and S fertilization upto 120 and 60 kg ha^–1^, respectively. The highest nutrient use efficiency traits were recorded at 60 kg N ha^–1^ and 20 kg S ha^–1^. It was concluded that 120 kg N ha^–1^ and 40 kg S ha^–1^ can be proposed for *T. minuta* as a result of agronomic responses, which serves as a sustainable means of cropping.

## Introduction

Wild marigold (*Tagetes minuta* L.) is the most widely cultivated species (Family Asteraceae) which grows in moist and dry areas of the tropical, subtropical and temperate region within an altitude ranging from 1000 to 2500 m amsl (Singh et al., 2003). It is a strongly scented annual herb with erect and highly branched stem and it has an affinity for disturbed sites and can colonize waste ground, roadsides, gardens, orchards, and vine yards. It has traditionally been used in diverse health problems such as colds, stomach ailments and breathing problems, and act as sedative, anti-septic, insecticidal, anti-parasitic and antispasmodic agent (Walia and Kumar, 2020). Food and flavoring, perfumery, pharmaceutical, and agrochemical industries are the major user of its essential oil (EO). Its EO has *Z*-β-ocimene, dihydrotagetone, (*E and Z*) tagetone and (*E and Z*) ocimenones as the major compounds (Walia et al., 2020). EO with higher percentage of *Z-β-*ocimene (35–50%) has high rate in international market. During 2016, its EO has world annual production of about 15 tons (Cornelius and Wycliffe, 2016) and its EO market is expected to reach 11.8 USD million by 2025 with a CAGR of 7.0% from 2020 to 2025 (as per EO market report 2020).

Latest studies showed that wild marigold may be a profitable EO crop for the western Himalaya and probably in the north India as this crop remains unaffected by biotic and abiotic factors and the essential oil obtained from hilly region are rich in ocimene and dihydrotagetone which are the major compounds used in perfumery and flavor industries (Rathore et al., 2018). Plant fertilization in *Tagetes* was not yet examined in the western Himalayas, however, previous studies on plant nutrients have shown a substantial effect in general (Sunitha and Hunje, 2010; Negahban et al., 2014; Chand et al., 2015) and nitrogen (N) fertilization (Omidbaigi et al., 2008; Singh et al., 2008; Singhal and Sharma, 2010) on wild marigold production and quality. Sulfur (S) is an important macronutrient in plants due to its important function in synthesis of proteins, as it is an important part of cysteine and methionine amino acids and coenzymes (Marschner, 1999). From many years’ deficiency of S in crops with high S requirement has been a main concern worldwide (Etienne et al., 2018). Soil receives S with rain and through fertilizer application of NPK, but recently highly pure fertilizers are in use with negligible S, leading to continuous removal of S without any supply making soil S deficient (Riley et al., 2002). S deficiency also affects the N uptake further reducing yield and quality of plants (Srinivasarao et al., 2008). Earlier studies showed that addition of S increased yield and EO quality in mint (*Mentha arvensis* L.), dragonhead (*Dracocephalum moldavica* L.) and sweet basil (*Ocimum basilicum* L.) (Aziz et al., 2010; Kumar et al., 2010; Oliveira et al., 2014). For wild marigold S requirement is unclear or did not appear in the literature.

Fertilizer occupies an important role in increasing biomass yield of wild marigold, with the percentage increase of even more than 50% (Prakasa Rao et al., 2000; Singh and Rao, 2005; Singh et al., 2008) and improves livelihood of people’s. However, while ensuring agricultural production, there are issues with unnecessary and blind use of chemical fertilizers, which raise the risk of contamination in the atmosphere. It is therefore imperative to limit unnecessary inputs of fertilizers and ensure their efficient supply, both of which facilitate sustainable agricultural growth. The level of fertilization should be realistically designed to minimize blind application of fertilizers and should depend on soil conditions, yield capacity and systematic control of nutrients in different regions. Therefore, it is imperative to standardize the doses of fertilizers and increase the quality of usage, with the goal of encouraging sustainable development in *T. minuta*.

This study assessed the hypothesis that yield and EO composition of wild marigold will be significantly influenced by standardized doses of N and S. In wild marigold, major components of EO viz. *Z-β-*ocimene, dihydrotagetone and tagetone are of great interest to different industries; hence, in such systems oil yield and quality are more important. So, this study standardize the doses of N and S and improve nutrient use efficiency, aiming to increase total *Tagetes* production (*viz.* herbage yield, EO yield, and composition), and promote the sustainable development.

## Materials and Methods

### Site Description and Field Procedures

**FIGURE 1:**
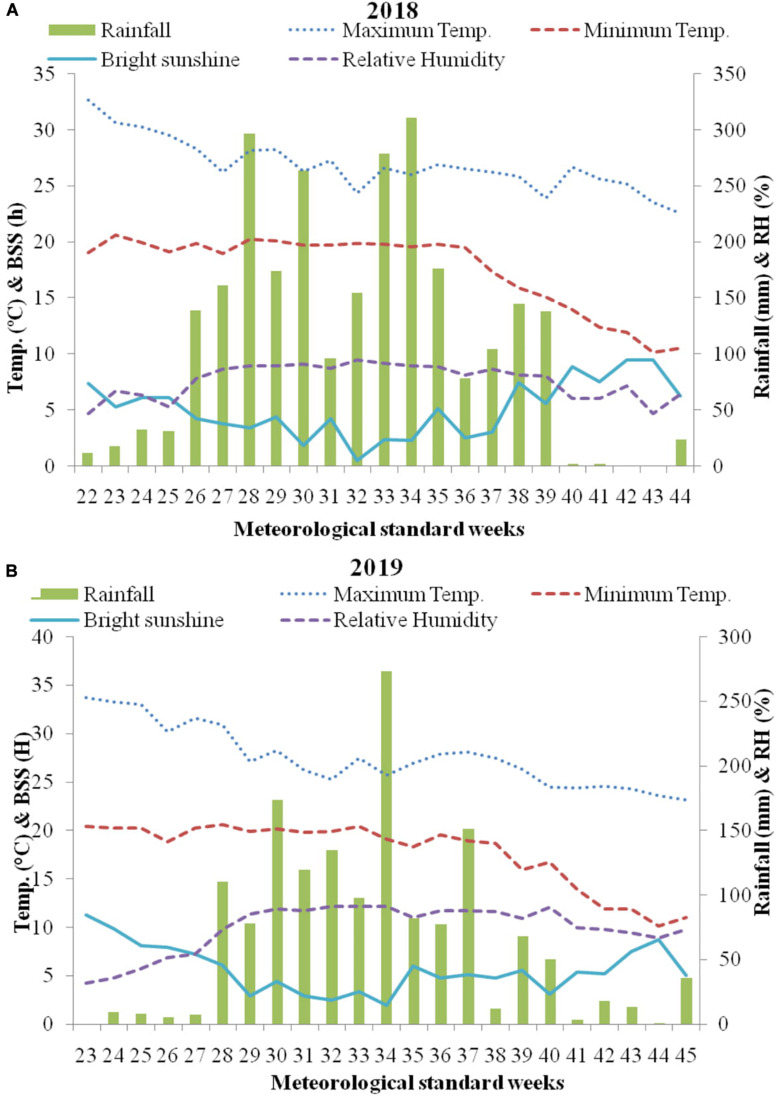
Weekly mean climatological data (temperature, rainfall, sunshine hours, and relative humidity) during the growing season **(A)** 2018 and **(B)** 2019 at Palampur, India. BSS, Bright sunshine hours; RH, Relative humidity.

This study was conducted at CSIR-Institute of Himalayan Bioresource Technology (CSIR-IHBT, Palampur, India; 32°11′N, 76°56′E; 1325 m amsl) during 2018 and 2019 with mean temperature and rainfall of 18°C and 250 cm, respectively. Mean weekly data during crop growing season obtained from agro-meteorological advisory, crop weather outlook (Anonymous, 2019), are detailed in Figure 1. The soil traits were: texture silty clay; acidic pH (5.26); low electrical conductivity (0.05 mmhos cm^–1^); low organic C (0.70%); low available nitrogen (112.37 kg ha^–1^); low available phosphorus (8.80 kg ha^–1^); high available potassium (394.00 kg ha^–1^) and very low available sulfur (7.64 kg ha^–1^). During March–April of both the years, seedbed preparation was done. Before seed sowing, farm yard manure (FYM) at 15t/ha was incorporated into the soil. During both the years pre sowing fertilization include 60 kg ha^–1^ P_2_O_5_ (triple superphosphate) and 40 kg ha^–1^ K_2_O (muriate of potash). Seeds of HIM GOLD (IHBT.MARIGOLD.I) an improved variety of *T. minuta*, developed by CSIR-Institute of Himalayan Bioresource Technology, Palampur, India were planted at rate of 3 kg ha^–1^, at 60 cm row spacing in the first week of June during both the years. To maintain homogeneity and to allow adequate space for the remaining plants to grow efficiently crop thinning was done in all the plots, keeping eight plants per row.

### Treatments and Data Collection

Experimental design was factorial randomized block design (RBD) during 2018 and 2019 with two factors, i.e., four N (Control, 60, 90, and 120 kg ha^–1^) and four S levels (Control, 20, 40, and 60 kg ha^–1^). The experiment consists of 16 treatments with three replicates. During both the years, 1/3 dose of N and full dose of S were given as pre sowing fertilization through urea (N 46%) and elemental S (S 99%). Top dressing consists of N through urea 1/3 dose each at stem elongation and flowering times, as per the treatments.

At harvest, growth parameters *viz*., plant height and numbers of branches were recorded from five selected plant from each plot. Fresh biomass was calculated by manually harvesting the plants in the month of October during both the years with the help of sickles at maturity stage 30 cm above the ground to minimize experimental error. The leaf-to-stem ratio was calculated on a fresh weight basis.

### Stomatal and Leaf Oil Gland Characteristics

To observe the morphological characteristics of the leaf, stomata and oil glands, surface topography of leaf sample were examined at full boom stage of crop during second cropping season by Scanning Electron Microscope (SEM S-3400 N) of Hitachi, Japan. On aluminum stubs fresh leaf samples were mounted using double-sided carbon tape and then coated with a thin layer of gold with the help of sputter-coater at a vacuum of 10 Pa for 10 s to provide electrical conductivity. The sample stub was further loaded to specimen holder which was then connected to specimen exchange chamber in SEM. The images were captured at desired magnification at an accelerating filament voltage of 30 Kv. At least 3 leaflets treatment^–1^ and 1 leaflet plant^–1^ were used to observe the distribution and density of distinctive structures, averaging the density on 1 mm^2^ areas and length and width in micrometer (μm) on the micrograph obtained for each leaf.

### Essential Oil Extraction and Identification of Compounds by GCMS and GC

Essential oil was extracted from fresh plant material of *T. minuta* through hydro distillation in Clevenger equipment upto 4 h. The EO content (v/w %) and EO yield was calculated. EO obtained was analyzed gas chromatography (GC) and Gas chromatography–Mass spectrometry (GC–MS) analyses as per the procedure mentioned in Walia et al. (2020).

### NPKS Analysis in Plant Parts and Soil

For studying the outcome of experiment on nutrient (NPKS) uptake dry samples of leaf, flower, and stem from each treatment was prepared. Samples were digested with concentrated H_2_SO_4_ for N estimation and a mixture of concentrated H_2_SO_4_ and perchloric acid (5:1) for P, K, and S estimation. N and K was estimated with automatic nitrogen analyzer Kel Plus; P and S with spectrophotometer (model T 90 + UV/vis, PG Instrument Ltd.), while K with flame photometer (model BWB XP, UK Ltd., United Kingdom) as per method given by Yaduvanshi et al. (2009). In order to study the outcome of N and S on available nutrients (NPKS), samples of soil were obtained after the crop harvesting of both cropping season from surface layer of 0–15 cm. Soil available N was estimated with the help of an automatic N analyzer unit by Kel Plus. Available P, K (BWB XP, UK Ltd., United Kingdom) and S were measured according to the method given by Yaduvanshi et al. (2009), respectively.

### Nutrient Use Efficiency Traits

Nutrient recovery (NR) and nutrient use efficiency (NUE) of the crop were find out through formulas given by Fageria et al. (2011).

Agronomic efficiency (AE kg kg^–1^) described as yield per unit of nutrient applied.

$$\text{AE}\,\left(\mathrm{kg}/\mathrm{kg}\right)=\frac{\mathrm{Yf}-\mathrm{Yu}}{\text{Na}}$$

Where, Yf: yield in fertilized plots; Yu: yield in unfertilized plots; Na: nutrient applied.

Apparent recovery efficiency (ARE%) is nutrient uptake per unit of nutrient applied.

$$\text{ARE}\left(\%\right)=\frac{\mathrm{Nf}-\mathrm{Nu}}{\text{Na}}\times 100$$

Where, Nf: total nutrient uptake of the fertilized plot, Nu: total nutrient uptake of unfertilized plot; Na: quantity of nutrient applied.

Agro-physiological efficiency (APE kg kg^–1^) determined as

$$\text{APE}\,\left(\mathrm{kg}/\mathrm{kg}\right)=\frac{\mathrm{Yf}-\mathrm{Yu}}{\mathrm{Nf}-\mathrm{Nu}}$$

Where, Yf: yield in fertilized plots; Yu: yield in unfertilized plots; Nf: total nutrient uptake of the fertilized plot; Nu: total nutrient uptake of unfertilized plot.

### Statistical Analysis

The data recorded were checked for homogeneity of variance prior to analyzing the data for different parameters through analysis of variance (ANOVA) technique for factorial Randomized Block Design. Least significant difference (LSD) values were determine at *P* = 0.05 to the find the significant disparity between treatment means. A second degree- polynomial regression model was also established between fertilizer level of N and S, and yield parameters (biomass yield and essential oil yield) of *Tagetes*. Agronomic traits and essential oil composition were subjected to the principal component analysis (PCA) to understand those which were largely influenced by the treatments. PAST3 software was used for principal component analysis.

## Results

### Growth and Yield Parameters

The effect of cropping season was significant on growth parameters (Table 1). Statistically higher plant height (49.92, 145.89, and 243.15 cm) and number of branches plant^–1^ (14.71, 16.75, and 18.43) at 60, 90 DAS and harvest, respectively, were recorded during 2018 as compared to 2019 (Table 2). Despite the lowest growth parameters, significantly higher leaf + flower biomass (74.80 q ha^–1^), stem biomass (113.24 q ha^–1^), total biomass (188.03 q ha^–1^), and EO yield (93.91 kg ha^–1^) were found during 2019 than 2018 (Table 3). The second cropping year (2019), registered 17.79% and 26.29% higher total biomass and oil yield, respectively, than the first cropping year (2018).

**TABLE 1:** Analysis of variance for effect of cropping year and nutrient application on growth and yield traits of *T. minuta*.

Source of variation	df	Plant height			Number of branches plant^–1^			Leaf + flower biomass (q ha^–1^)	Total above ground biomass (q ha^–1^)	Leaf + flower/stem ratio	EO content (%)	EO yield (kg ha^–1^)
			
		60 DAS	90 DAS	At Harvest	60 DAS	90 DAS	At Harvest					
Cropping year (Y)	1	**	**	**	**	**	**	**	**	**	**	**
Nitrogen levels (N)	3	*	**	**	**	ns	ns	**	**	*	**	**
Sulfur levels (S)	3	*	**	**	*	*	ns	**	**	*	**	**
Y × N	3	ns	**	ns	ns	ns	ns	ns	ns	ns	ns	ns
Y × S	3	*	*	ns	ns	ns	ns	ns	ns	ns	ns	ns
N × S	9	ns	ns	**	ns	**	*	**	**	ns	ns	**
Y × N × S	9	**	**	ns	**	ns	ns	ns	ns	ns	ns	ns
Error	62											
CV	–	12.23	13.12	11.08	16.88	16.64	12.58	14.47	12.04	9.43	6.23	7.07

* and ** represent significant relationship at *P* ≤ 0.05 and *P* ≤ 0.01, respectively. ns, non-significant; DAS, days after sowing.

**TABLE 2:** Effect of year and fertilization levels on growth parameters of *T. minuta* at different growth stages.

Treatment	Plant height (cm)			Number of branches plant^–1^		
		
	60 DAS	90 DAS	At harvest	60 DAS	90 DAS	At harvest
**Cropping year**						
2018	49.92^*a*^	145.89^*a*^	243.15^*a*^	14.71^*a*^	16.75^*a*^	18.43^*a*^
2019	43.53^*b*^	139.39^*b*^	236.75^*b*^	8.36^*b*^	9.93^*b*^	11.89^*b*^
**Nitrogen level**						
Control (N0)	42.50^*d*^	126.18^*d*^	225.15^*d*^	9.88^*d*^	13.05^*ns*^	14.84^*ns*^
60 kg/ha (N1)	46.99^*abc*^	140.15^*c*^	236.31^*c*^	11.05^*c*^	13.58^*ns*^	15.02^*ns*^
90 kg/ha (N2)	48.65^*ab*^	152.13^*a*^	246.86^*b*^	12.74^*a*^	13.25^*ns*^	15.15^*ns*^
120 kg/ha (N3)	48.77^*a*^	152.09^*ab*^	251.46^*a*^	12.49^*ab*^	13.47^*ns*^	15.64^*ns*^
**Sulfur level**						
Control (S0)	42.40^*d*^	134.31^*d*^	233.65^*d*^	10.18^*cd*^	13.20^*abc*^	15.08^*ns*^
20 kg/ha (S1)	46.65^*abc*^	140.01^*c*^	238.85^*c*^	11.50^*abc*^	14.34^*a*^	15.70^*ns*^
40 kg/ha (S2)	48.89^*ab*^	148.15^*a*^	242.06^*b*^	12.01^*ab*^	12.49^*bcd*^	14.47^*ns*^
60 kg/ha (S3)	48.97^*a*^	148.07^*ab*^	245.23^*a*^	12.46^*a*^	13.33^*ab*^	15.40^*ns*^

*Means within each column with similar letter are not significantly different at the 5% probability level. DAS, Days after sowing; ns, non-significant.*

**TABLE 3:** Effect of year and fertilization levels on biomass and essential oil yield of *T. minuta*.

Treatment	Leaf + flower biomass (q ha^–1^)	Total biomass (q ha^–1^)	Leaf + flower/stem ratio	Essential oil content (%)	Essential oil yield (kg ha^–1^)
**Cropping year**					
2018	60.52^*b*^	159.62^*b*^	0.61^*b*^	0.55^*b*^	74.36^*b*^
2019	74.80^*a*^	188.03^*a*^	0.66^*a*^	0.59^*a*^	93.91^*a*^
**Nitrogen level**					
Control (N0)	61.00^*d*^	160.62^*d*^	0.61^*d*^	0.50^*d*^	68.02^*d*^
60 kg/ha (N1)	66.94^*c*^	171.82^*c*^	0.64^*abc*^	0.55^*bc*^	79.97^*c*^
90 kg/ha (N2)	70.00^*b*^	178.98^*b*^	0.65^*ab*^	0.57^*b*^	86.48^*b*^
120 kg/ha (N3)	72.70^*a*^	183.89^*a*^	0.66^*a*^	0.65^*a*^	102.09^*a*^
**Sulfur level**					
Control (S0)	63.99^*d*^	167.59^*d*^	0.62^*cd*^	0.52^*d*^	74.91^*d*^
20 kg/ha (S1)	66.69^*c*^	172.16^*c*^	0.63^*bc*^	0.57^*abc*^	83.98^*c*^
40 kg/ha (S2)	69.28^*ab*^	176.67^*b*^	0.65^*ab*^	0.59^*a*^	89.06^*a*^
60 kg/ha (S3)	70.67^*a*^	178.90^*a*^	0.66^*a*^	0.58^*ab*^	88.60^*ab*^

*Means within each column with similar letter are not significantly different at the 5% probability level.*

Among the nutrient levels, N at 120 kg ha^–1^ and S at 60 kg ha^–1^ produced taller plants (48.77 cm and 48.97 cm, respectively) at 60 DAS which remained statically at par with other two doses of N and S than control. At 90 DAS, significantly higher plants were produced with N at 90 kg ha^–1^ (152.13 cm) and S at 40 kg ha^–1^ (148.15 cm) which were in line with N at 120 kg ha^–1^ and S at 60 kg ha^–1^, respectively, than control. Significantly taller plants were recorded with 120 kg N ha^–1^ (251.46 cm) and 60 kg S ha^–1^ (245.23 cm) than other treatments at harvest. Higher number of branches plant^–1^ were produced by 90 kg N ha^–1^ (12.74) and 60 kg S ha^–1^ (12.46) at 60 DAS than other treatments, whereas former was in line with N at 120 kg ha^–1^. No significant effect was noticed at 90 DAS and harvest on number of branches plant^–1^ with N and S application except for S at 90 DAS, which was maximum at 20 kg ha^–1^ and behaved statistically similar with S at 60 kg ha^–1^ (Table 2).

In case of yield parameters, significantly higher leaf + flower biomass (72.70 q ha^–1^), stem biomass (111.20 q ha^–1^), total biomass (183.89 q ha^–1^), and EO yield (102.09 kg ha^–1^) were found with N at 120 kg ha^–1^ as compared to other treatments. However, S at 60 kg S ha^–1^ observed maximum leaf + flower biomass (70.67 q ha^–1^), stem biomass (108.22 q ha^–1^), and total biomass (178.90 q ha^–1^), when former two parameters were similar with S at 40 kg ha^–1^. EO yield (89.06 kg ha^–1^) was statistically higher at 40 kg S ha^–1^ than control, but in line with 60 kg S ha^–1^ (Table 3). In this investigation, the effects of cropping season and nutrient application on EO content (%) were significant (*P* ≥ 0.05) (Table 3). Year 2019 recorded significantly higher EO content (0.59%) than 2018 (0.55%). Irrespective of years, N application increased the EO content by 30.00% at 120 kg ha^–1^ than control. However, EO content (0.59%) at 40 kg ha^–1^ was statistically higher with 13.46% than control, but found similar with remaining S doses.

### Correlation and Regression Analysis

**FIGURE 2:**
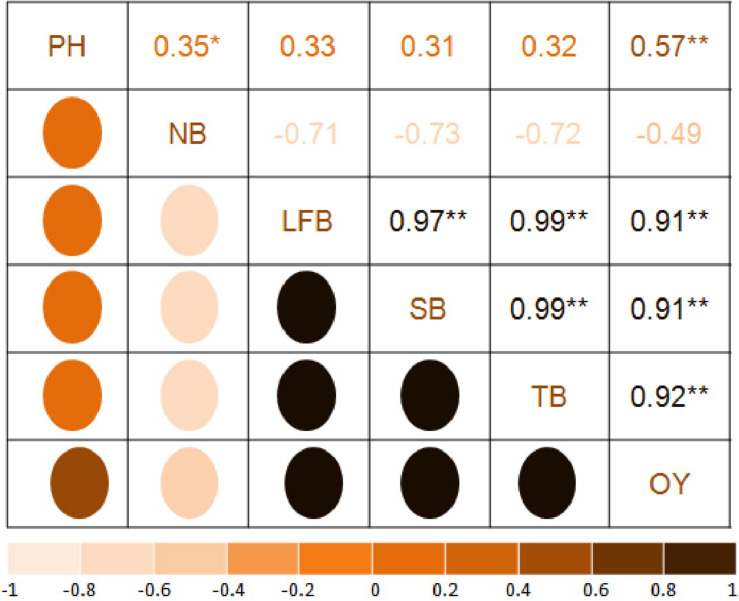
Correlation analysis of growth and yield parameters. PH, plant height; NB, number of branches per plant; LFB, leaf+flower biomass; SB, stem biomass; TB, total biomass. OY, oil yield. The mean values of the 2 years pooled data of the corresponding treatments are used (where N1 = N2 = 16); * and ** Indicate that the corresponding values are significant at *P* = 0.05 and *P* = 0.01, respectively.

The correlation matrix of *T. minuta* (Figure 2) showed positive and negative correlation of EO yield with plant height (*r* = 0.57) and number of branches (*r* = −0.49), respectively. Among biomass significantly (*P* = 0.01) positive correlation of EO yield was observed with leaf + flower biomass (*r* = 0.91), stem biomass (*r* = 0.91), and total biomass (*r* = 0.92). Total biomass showed positive (*P* = 0.01) correlation with leaf + flower (*r* = 0.99) and stem biomass (*r* = 0.99), and negative with number of branches (*r* = −0.72). Leaf + flower and stem biomass showed positive correlation with plant height (*r* = 0.33 and *r* = 0.31, respectively) and negative with number of branches (*r* = −0.71 and *r* = −0.73, respectively).

**FIGURE 3:**
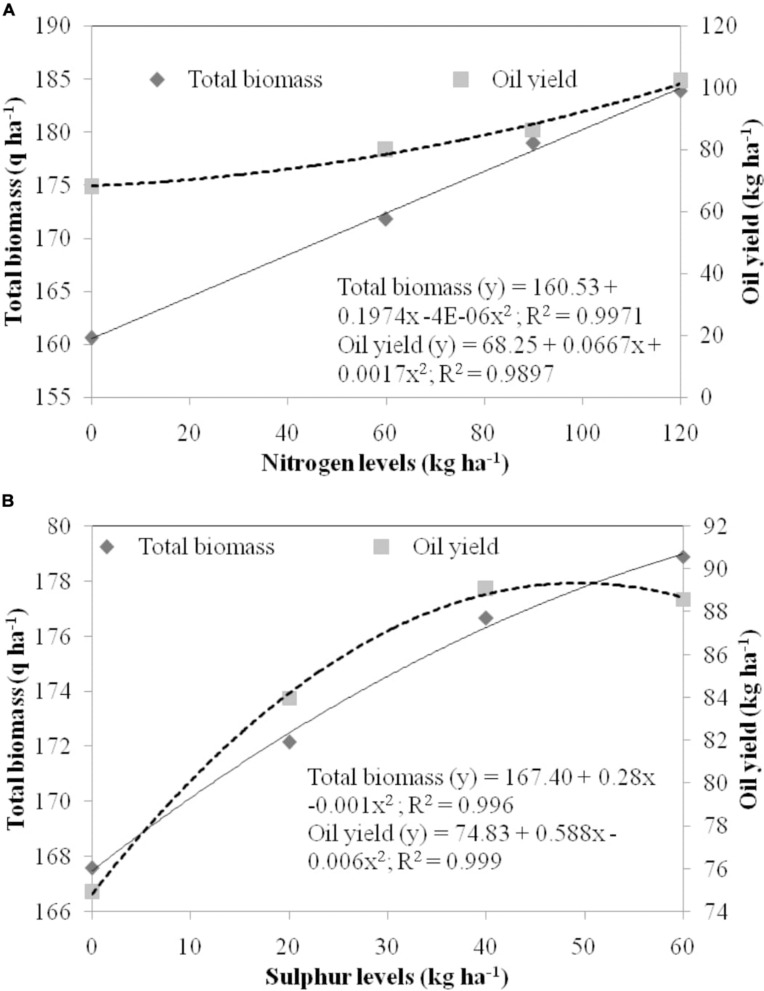
Regression equation between independent variable, **(A)** levels of nitrogen **(B)** levels of sulfur and dependent variables, i.e., total biomass (q ha^– 1^) and oil yield (kg ha^– 1^). The doses of nitrogen and sulfur are represented in the primary *X* axis. Total biomass and oil yield are presented in the primary *Y* axis and secondary *Y* axis, respectively.

Regression equations among independent variables N and S and dependent variables total biomass and EO yield were created (Figure 3). The magnitude of total biomass and EO yield increased with increase in N dose and was found highest in 120 kg N ha^–1^ (Figure 3A). A strong relationship was observed for N doses with total biomass and EO yield with equation *y* = 160.53 + 0.1974x −4E-06x^2^ (*R*^2^ = 0.997; *P* = 0.01) and *y* = 68.25 + 0.0667x + 0.0017x^2^ (*R*^2^ = 0.989; *P* = 0.01), respectively. Among S doses, total biomass enhanced with higher doses of S, while EO yield enhanced till 40 kg S ha^–1^ with a decline thereafter (Figure 3B). Therefore, a strong relationship was established by levels of S with total biomass (167.40 + 0.28x −0.001x^2^; *R*^2^ = 0.996; *P* = 0.01) and EO yield (*y* = 74.83 + 0.588x – 0.006x^2^; *R*^2^ = 0.999; *P* = 0.01).

### Stomatal and Leaf Oil Glands Characteristics

**FIGURE 4:**
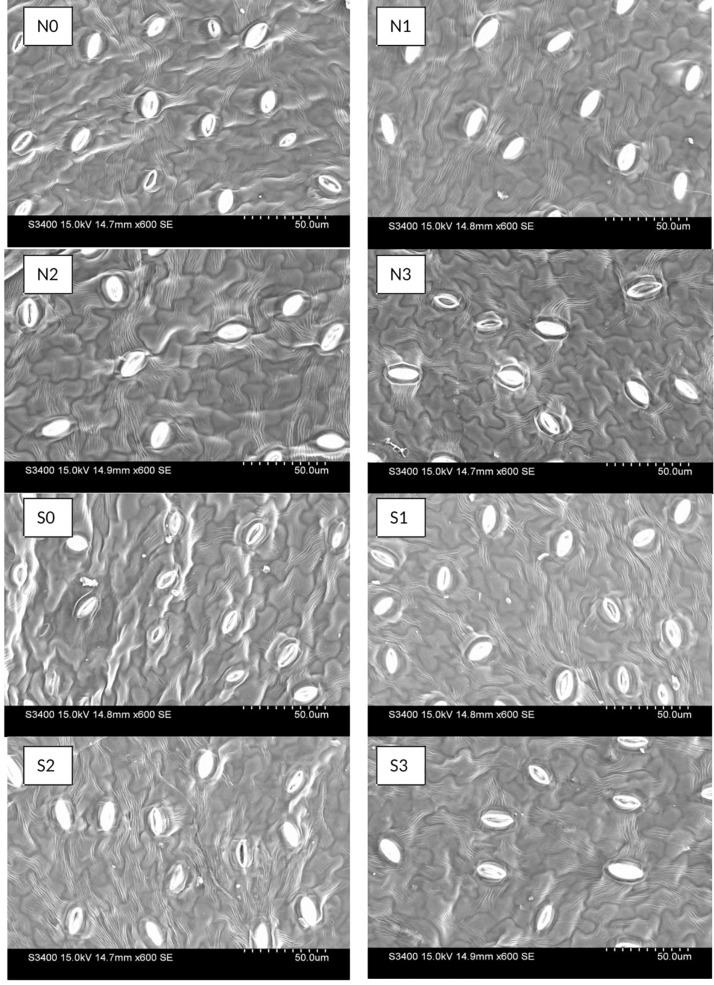
Effect of nitrogen (N) and sulfur (S) fertilization levels on stomatal characteristics. N0, N1, N2, and N3 are the level of nitrogen @ 0, 60, 90, and 120 kg ha^– 1^, respectively, while S0, S1, S2, and S3 are representing the level of sulfur @ 0, 20, 40, and 60 kg ha^– 1^, respectively.

Electron-microscopic observations of abaxial surface of leaves showed significant result on stomatal densities and dimensions (stomata length and pore length) with the use N and S fertilizers (Figure 4). The decrease in stomatal density seems to be consistent with increasing N and S doses, and was found higher in control than other treatments. In case of stomatal length, 120 kg N ha^–1^ and 40 kg S ha^–1^ observed higher (*P* = 0.05) length than control. Stomatal aperture did not follow any particular trend (Table 4).

**TABLE 4:** Effect of fertilization levels on stomata and oil gland parameters of *T. minuta* during 2019.

Treatment	Stomata			Oil gland		
		
	Number (per mm^2^)	Length (μ m)	Aperture (μ m)	Number (per mm^2^)	Length (μm)	Width (μm)
**Nitrogen level**						
Control (N0)	42.08^*a*^	19.13^*bcd*^	15.64^*a*^	1.33^*ns*^	248.05^*a*^	167.20^*a*^
60 kg/ha (N1)	41.75^*ab*^	19.20^*bc*^	11.39^*cd*^	1.58^*ns*^	195.12^*bc*^	144.62^*b*^
90 kg/ha (N2)	38.00^*c*^	20.30^*b*^	13.16^*b*^	1.71^*ns*^	190.91^*cd*^	142.20^*bc*^
120 kg/ha (N3)	34.25^*d*^	22.50^*a*^	12.77^*bc*^	1.71^*ns*^	199.12^*b*^	137.50^*d*^
**Sulfur level**						
Control (S0)	48.50^*a*^	18.33^*d*^	11.72^*cd*^	1.08^*bc*^	215.83^*a*^	139.12^*d*^
20 kg/ha (S1)	32.75^*d*^	21.38^*a*^	14.65^*a*^	1.33^*b*^	200.23^*cd*^	148.95^*b*^
40 kg/ha (S2)	36.41^*bc*^	20.89^*ab*^	14.10^*ab*^	1.96^*a*^	214.97^*ab*^	156.50^*a*^
60 kg/ha (S3)	38.41^*b*^	20.54^*abc*^	12.50^*c*^	1.96^*a*^	202.16^*c*^	146.95^*bc*^

*Means within each column with similar letter are not significantly different at the 5% probability level.*

**FIGURE 5:**
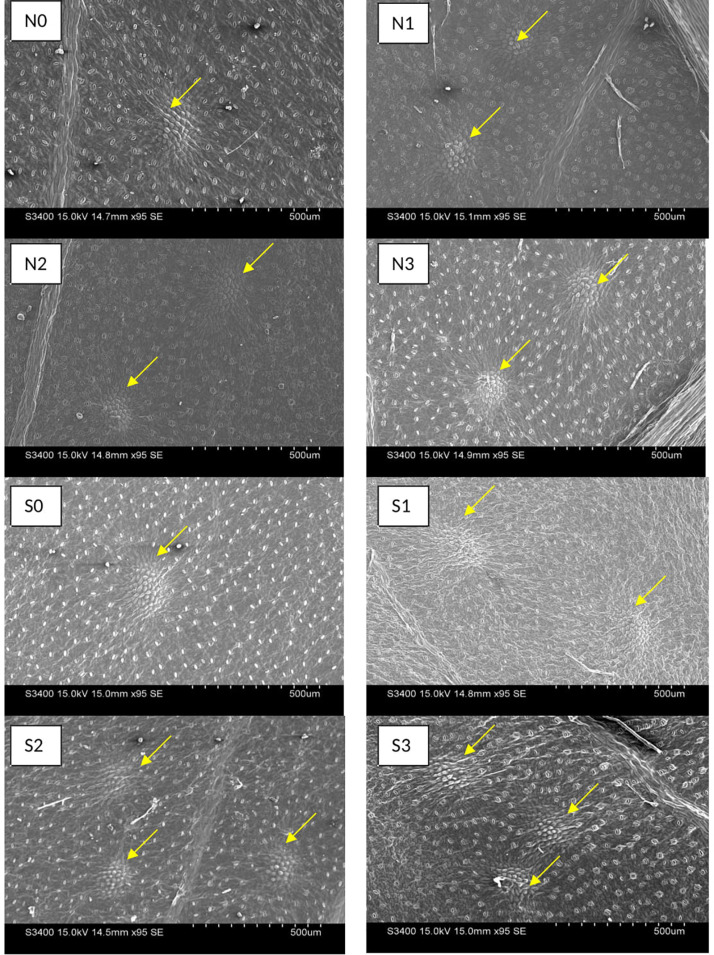
Effect of nitrogen (N) and sulfur (S) fertilization levels on leaf oil glands characteristics. N0, N1, N2, and N3 are the level of nitrogen @ 0, 60, 90, and 120 kg ha^– 1^, respectively, while S0, S1, S2, and S3 are representing the level of sulfur @ 0, 20, 40, and 60 kg ha^– 1^, respectively.

The oil glands were observed on abaxial surface of *T. minuta* leaves (Figure 5). N and S dose recorded marked impact on oil gland characteristics in *T. minuta*. The population of oil glands appears more in S treated plants as compared to control, however, no significant effect was observed with N fertilization (Table 4). The plants with 60 and 40 kg S ha^–1^ reported about 81.48% higher leaf oil glands than control. In case of length and width of oil glands, leaves from control plots recorded significantly higher values in comparison to other doses of both N and S.

### N × S Interaction

Total biomass and EO yield recorded significant interaction of N and S fertilization treatment during both the years (Table 5). Among N fertilization levels, highest total biomass yield was observed in N3 (120 kg N ha^–1^) than other levels during 2018 and 2019. Among S fertilization levels, S3 (60 kg S ha^–1^) showed enhanced total biomass yield than other treatments but was in line with S2 (40 kg S ha^–1^) during 2019. Among all treatment combinations N3S3, gave highest total biomass yield during 2018 and 2019, but remained statistically similar with N3S2 during 2019. In case of EO yield, N3 (120 kg N ha^–1^) recorded highest EO yield among N levels during both the years. Among S fertilization levels, S2 (40 kg S ha^–1^) recorded enhanced EO yield in 2018 and 2019, but were statistically similar to S3 (60 kg S ha^–1^) during both the years. Among all treatment combinations N3S2 recorded highest EO yield during 2018 and 2019, but were statistically in line with N3S3 during both the years. It was concluded that N3S2, i.e., 120 kg N ha^–1^ and 40 kg S ha^–1^ can be suggested for *T. minuta* based on biomass and EO yield response.

**TABLE 5:** Interaction effect of N and S fertilization levels on yield of *T. minuta* during 2018 and 2019.

	**Total biomass yield (q ha^–1^)**									
	
	**2018**					**2019**				
		
**Treatment**	**S0**	**S1**	**S2**	**S3**	**Mean (S)**	**S0**	**S1**	**S2**	**S3**	**Mean (S)**
N0	132.2^*o*^	137.9^*mn*^	139.2^*mn*^	139.9^*lm*^	137.3^*D*^	156.3^*nop*^	164.1^*mn*^	163.2^*mno*^	166.1^*jklm*^	162.4^*D*^
N1	141.4^*l*^	144.8^*k*^	150.2^*hi*^	150.2^*f*^	146.6^*C*^	171.0^*fghijkl*^	172.7^*fghij*^	176.6^*defg*^	175.8^*fgh*^	174.0^*C*^
N2	151.8^*fg*^	150.6^*fgh*^	156.2^*cde*^	156.9^*c*^	153.9^*B*^	178.0^*cdef*^	174.5^*fghi*^	183.7^*cd*^	184.5^*bc*^	180.2^*B*^
N3	148.3^*hij*^	156.9^*cd*^	163.0^*b*^	163.6^*a*^	157.9^*A*^	172.1^*fghijk*^	183.6^*cde*^	191.4^*ab*^	193.9^*a*^	185.3^*A*^
Mean (N)	143.4^*D*^	147.6^*C*^	152.1^*B*^	152.6^*A*^		169.3^*D*^	173.7^*C*^	178.7^*AB*^	180.1^*A*^	

	**Essential oil yield (kg ha^–1^)**									
	
	**2018**					**2019**				
		
**Treatment**	**S0**	**S1**	**S2**	**S3**	**Mean (S)**	**S0**	**S1**	**S2**	**S3**	**Mean (S)**

N0	48.1^*op*^	54.9^*lmno*^	59.2^*klm*^	60.3^*ijkl*^	55.6^*D*^	62.1^*p*^	70.7^*lmno*^	75.0^*lmn*^	77.3^*jkl*^	71.3^*D*^
N1	58.6^*klmn*^	66.5^*efghij*^	70.7^*defgh*^	66.5^*defghi*^	65.6^*C*^	76.7^*jklm*^	85.1^*efghi*^	89.0^*defgh*^	83.8^*ghij*^	83.7^*C*^
N2	65.4^*hijk*^	73.4^*defg*^	73.6^*def*^	73.7^*de*^	71.5^*B*^	82.9^*hijk*^	90.9^*defg*^	92.6^*de*^	92.8^*d*^	89.8^*B*^
N3	73.9^*d*^	82.6^*c*^	92.1^*a*^	90.6^*ab*^	84.8^*A*^	91.5^*def*^	102.7^*c*^	114.6^*a*^	113.9^*ab*^	105.7^*A*^
Mean (N)	61.5^*D*^	69.3^*C*^	73.9^*A*^	72.83^*AB*^		78.3^*D*^	87.4^*C*^	92.8^*A*^	92.0^*AB*^	

*Means within each column with similar letter are not significantly different at the 5% probability level.*

### Essential Oil Composition

The outcome of various treatments was assessed on EO constituents of *T. minuta* (Table 6). Overall, analysis of EO identifies 10 constituents accounting for 91.17–94.36% of the total percentage. The GC-FID reported *Z-β-*ocimene (34.18–42.59%), dihydrotagetone (18.83–24.73%), *E*-tagetone (0.80–1.17%), *Z*-tagetone (8.54–9.68%), *Z*-ocimenone (6.01–8.42%), and *E*-ocimenone (8.43–12.85%) as major constituents of EO. *T. minuta* EO presented acyclic monoterpenes (87.05–90.86%) as one of the major chemical group of terpenes.

**TABLE 6:** Effect of year and fertilization levels on essential oil composition of *T. minuta*.

	Essential oil compounds (%)													
	
Treatment	Sabinene	Limonene	Z- ocimene	Dihydrotagetone	Allo-ocimene	E- tagetone	Z-tagetone	Z-ocimenone	E-ocimenone	*Trans* Caryophyllene	Acyclic monoterpenes	Cyclic monoterpenes	Bicyclic monoterpenes	Total
Litt RI	969	1024	1032	1046	1128	1139	1148	1226	1235	1408				
Exp. RI	972	1029	1033	1052	1121	1146	1154	1234	1242	1394	**Grouped components**			
	
**Cropping season**														
2018	0.33^*b*^	3.03^*a*^	34.18^*b*^	24.73^*a*^	1.00^*a*^	1.17^*a*^	8.54^*b*^	8.42^*a*^	10.44^*b*^	0.29^*b*^	88.51^*b*^	3.03^*a*^	0.62^*b*^	92.16^*b*^
2019	0.79^*a*^	2.43^*b*^	41.75^*a*^	18.83^*b*^	0.88^*b*^	0.80^*b*^	9.68^*a*^	6.25^*b*^	11.37^*a*^	0.44^*a*^	89.58^*a*^	2.43^*b*^	1.23^*a*^	93.26^*a*^
**Nitrogen level**														
Control (N0)	0.47^*d*^	2.90^*b*^	34.44^*d*^	24.28^*a*^	0.92^*bc*^	0.95^*b*^	9.56^*a*^	8.08^*a*^	9.66^*c*^	0.33^*b*^	87.91^*c*^	2.90^*b*^	0.80^*d*^	91.62^*c*^
60 kg/ha (N1)	0.57^*b*^	2.50^*d*^	36.36^*c*^	20.27^*c*^	0.93^*b*^	0.92^*bc*^	9.02^*bc*^	7.43^*c*^	12.85^*a*^	0.43^*a*^	87.49^*d*^	2.50^*d*^	1.00^*a*^	91.43^*cd*^
90 kg/ha (N2)	0.68^*a*^	2.98^*a*^	38.47^*b*^	22.68^*b*^	0.99^*a*^	1.14^*a*^	9.21^*b*^	7.82^*b*^	9.26^*cd*^	0.27^*c*^	89.92^*b*^	2.98^*a*^	0.95^*b*^	93.43^*b*^
120 kg/ha (N3)	0.51^*c*^	2.55^*c*^	42.59^*a*^	19.90^*d*^	0.91^*bcd*^	0.92^*bc*^	8.67^*d*^	6.01^*d*^	11.85^*b*^	0.43^*a*^	90.86^*a*^	2.55^*c*^	0.94^*bc*^	94.36^*a*^
**Sulfur level**														
Control (S0)	0.59^*a*^	3.16^*a*^	35.14^*d*^	24.10^*a*^	0.99^*a*^	1.15^*a*^	9.63^*a*^	7.59^*ab*^	8.43^*d*^	0.34^*c*^	87.05^*d*^	3.16^*a*^	0.93^*bc*^	91.17^*d*^
20 kg/ha (S1)	0.55^*b*^	2.59^*c*^	36.21^*c*^	21.95^*c*^	0.90^*cd*^	0.85^*cd*^	8.96^*bc*^	7.41^*bc*^	11.23^*bc*^	0.39^*b*^	88.78^*c*^	2.59^*c*^	0.94^*b*^	92.31^*c*^
40 kg/ha (S2)	0.49^*c*^	2.70^*b*^	38.16^*b*^	22.25^*b*^	0.95^*b*^	1.03^*b*^	9.15^*b*^	7.69^*a*^	11.48^*b*^	0.32^*cd*^	89.49^*b*^	2.70^*b*^	0.81^*d*^	93.04^*b*^
60 kg/ha (S3)	0.59^*a*^	2.46^*d*^	42.35^*a*^	18.85^*d*^	0.91^*c*^	0.90^*c*^	8.71^*cd*^	6.63^*d*^	12.48^*a*^	0.41^*a*^	90.86^*a*^	2.46^*d*^	1.00^*a*^	94.34^*a*^

*Means within each column with similar letter are not significantly different at the 5% probability level.*

The percentage of EO constituents, i.e., dihydrotagetone, *E*-tagetone, and *Z*-ocimenone in 2019 reduced by 23.85, 31.62, and 25.77%, respectively, compared to 2018, however, percentage of *Z*-ocimene, *Z*- tagetone, and *E*- ocimenone increased by 22.14, 13.34, and 8.90%, respectively, during 2019 as compared to 2018. Nutrient fertilization treatments affected the percentage of main constitutes differently. N and S showed significant effect on *Z-β-*ocimene, and the maximum quantity (42.59 and 42.35%, respectively) was recorded with N (120 kg ha^–1^) and S (60 kg ha^–1^). These application rates recorded about 23.66 and 20.51% higher *Z-β-*ocimene, respectively, as compared to control. Dihydrotagetone, *Z*-tagetone, and *Z*-ocimenone in case of N levels, while dihydrotagetone, *E*-tagetone, and *Z*- tagetone in case of S levels reported significantly higher percentages in control as compared to other fertilization levels. Among N levels, the percentage of *E*-tagetone (1.14%) and *E*-ocimenone (12.85%) increased with 90 and 60 kg N ha^–1^, respectively, than rest of N treatments. Significantly higher quantity of *Z*-ocimenone (7.69%) and *E*-ocimenone (12.48%) was observed with S at 40 and 60 kg ha^–1^, respectively, than other S application rates. These application rates recorded 1.31 and 48.04% higher *Z*-ocimenone and *E*-ocimenone, respectively, as compared to control.

Among grouped components acyclic monoterpenes covered maximum area percentage as compared to cyclic and bicyclic monoterpenes (Table 6). N and S observed significant effect on acyclic monoterpenes, and the maximum quantity (90.86%) was recorded with N (120 kg ha^–1^) and S (60 kg ha^–1^). Total percentage area of EO also showed similar trend with the maximum quantity (94.36 and 94.34%, respectively) with N (120 kg ha^–1^) and S (60 kg ha^–1^).

### Principal Component Analysis (PCA)

**FIGURE 6:**
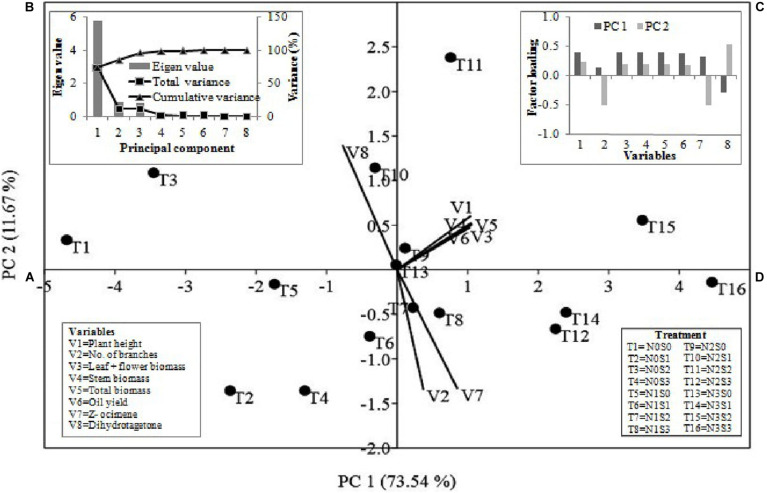
The multivariate analyses of mean value of growth parameters, yield and major compounds of essential oil were conducted through principal component analysis. PC1 and PC2 jointly explained the variations of 85.21% **(A–D)**. The loading values of variables and treatment combinations **(A)** are presented as vectors in the space of the PCA. **(B,C)** presented eigenvalues and loading scores of the variables with PC1 and PC2. N0, N1, N2, and N3 are the level of nitrogen @ 0, 60, 90, and 120 kg ha^–1^, respectively, while S0, S1, S2, and S3 are representing the level of sulfur @ 0, 20, 40, and 60 kg ha^–1^, respectively.

Principal component analysis was carried out using eight variables (six agronomic traits and two EO compounds) of *Tagetes* to know the relation between N and S doses and these variables (Figure 6). The statistics revealed that 85.21% of the total variations were jointly clarified by PC1 and PC2. Figure 6A represents the distribution of variables and treatment combinations. Among variables, except dihydrotagetone (V8), all variables [plant height (V1), branches (V2), flower + leaf biomass (V3), stem biomass (V4), total biomass (V5), EO yield (V6), and *Z-β-*ocimene (V7)] showed affirmative involvement in PC1. Similarly, in PC2, except for branches and *Z-β-*ocimene all other variables showed positive involvement (Figures 6A,C). The PCA also separated the treatment T11 (N2S2) and T15 (N3S2) by PC1 and PC2 and showed positive involvement in both PCs with strong relationships with most of the variables (Figure 6A). This study also showed that first three PCs with eigenvalues 5.85, 0.97, and 0.89, respectively, were most informative accounting for approximately 96.45% of the overall variance for the entire variables (Figure 6B).

### Plant Nutrient Concentration, Soil Available Nutrients and Nutrient Use Efficiency Traits

**FIGURE 7:**
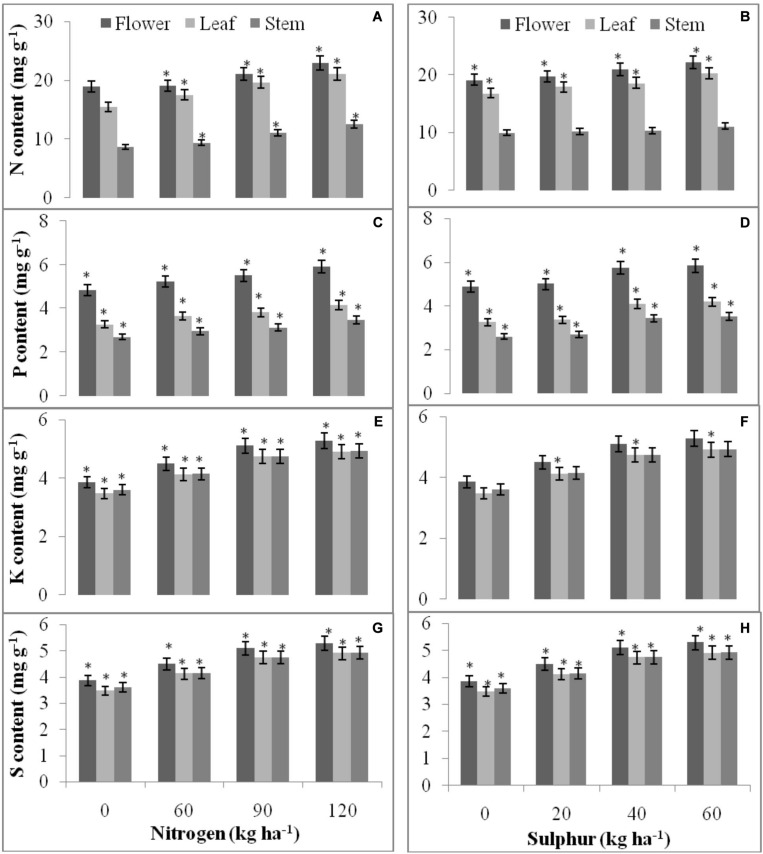
Spatial and temporal accumulation of N **(A,B)**, P **(C,D)**, K **(E,F)**, and S **(G,H)** in *T. minuta* flower, leaf, and stem as influenced by applied N and S fertilizers. The mean values of 2 years pooled data are presented. Within each bar, the asterisk (*) indicates significant differences among fertilizer levels at *P* = 0.05. Vertical bars indicate a mean standard error (±).

The data revealed significant (*P* = 0.05) relation between nutrients concentration (N/P/K/S mg g^–1^ dry plant tissue) in above ground parts of wild marigold and different fertilizer doses of N and S, except for N (stem) and K (flower and stem) concentration in case of S fertilization (Figure 7). Higher N dose (120 kg N ha^–1^) showed higher N (22.94, 21.02, and 12.50 mg g^–1^), P (5.91, 4.16, and 3.49 mg g^–1^), K (16.71, 25.49, and 23.91 mg g^–1^), and S (5.82, 5.45, and 5.46) concentration by *Tagetes* flower, leaf and stem, respectively, than lower dose (60 and 90 kg/ha). Concentration of nutrients among S levels followed similar results as that of N, with highest nutrient concentration at higher dose of S (60 kg S ha^–1^). S at 60 kg ha^–1^ observed 22.19, 20.26, and 11.09 mg g^–1^ N, 5.84, 4.19, and 3.53 mg g^–1^ P, 16.24, 24.37, and 23.11 K and 5.29, 4.91, and 4.93 mg g^–1^ S concentration in flower, leaf and stem, respectively, of *T. minuta*.

Significant effect was also observed on available soil nutrients due to different cropping years and nutrient doses, except for available P and K due to S application (Table 7). Second cropping year recorded a higher available N (195.53 kg ha^–1^), P (14.42 kg ha^–1^), K (438.93 kg ha^–1^), and S (12.62 kg ha^–1^) at harvest than first year. Among the nutrient applications, higher available N, P, and S (203.48, 14.21 cm, and 12.55 kg ha^–1^, respectively) were noticed in N at 120 kg ha^–1^ than other treatments, whereas available P was statistically similar with N at 90 kg ha^–1^ and N at 60 kg ha^–1^. No significant effect was show by N application on available K. Among S applications, significantly higher available N and S (213.98 and 13.22 kg ha^–1^, respectively) were found in S at 60 kg ha^–1^, where former was in line with S at 40 kg ha^–1^ than other treatments.

**TABLE 7:** Effect of year and fertilization levels on soil available nutrients.

Treatment	Available nutrients (kg ha^–1^)			
	
	Nitrogen	Phosphorus	Potassium	Sulfur
**Cropping season**				
2018	175.48^*b*^	12.51^*b*^	408.57^*b*^	10.70^*b*^
2019	195.53^*a*^	14.42^*a*^	438.93^*a*^	12.62^*a*^
**Nitrogen level**				
Control (N0)	173.94^*cd*^	12.25^*d*^	411.18^*ns*^	10.92^*cd*^
60 kg/ha (N1)	177.95^*bc*^	13.54^*abc*^	415.56^*ns*^	11.46^*bc*^
90 kg/ha (N2)	186.65^*b*^	13.86^*ab*^	432.27^*ns*^	11.70^*b*^
120 kg/ha (N3)	203.48^*a*^	14.21^*a*^	436.00^*ns*^	12.55^*a*^
**Sulfur level**				
Control (S0)	151.18^*d*^	13.40^*ns*^	427.61^*ns*^	10.19^*d*^
20 kg/ha (S1)	180.44^*c*^	13.75^*ns*^	406.62^*ns*^	11.17^*c*^
40 kg/ha (S2)	196.42^*b*^	13.34^*ns*^	435.13^*ns*^	12.06^*b*^
60 kg/ha (S3)	213.98^*a*^	13.36^*ns*^	425.64^*ns*^	13.22^*a*^

*Means within each column with similar letter are not significantly different at the 5% probability level.*

**FIGURE 8:**
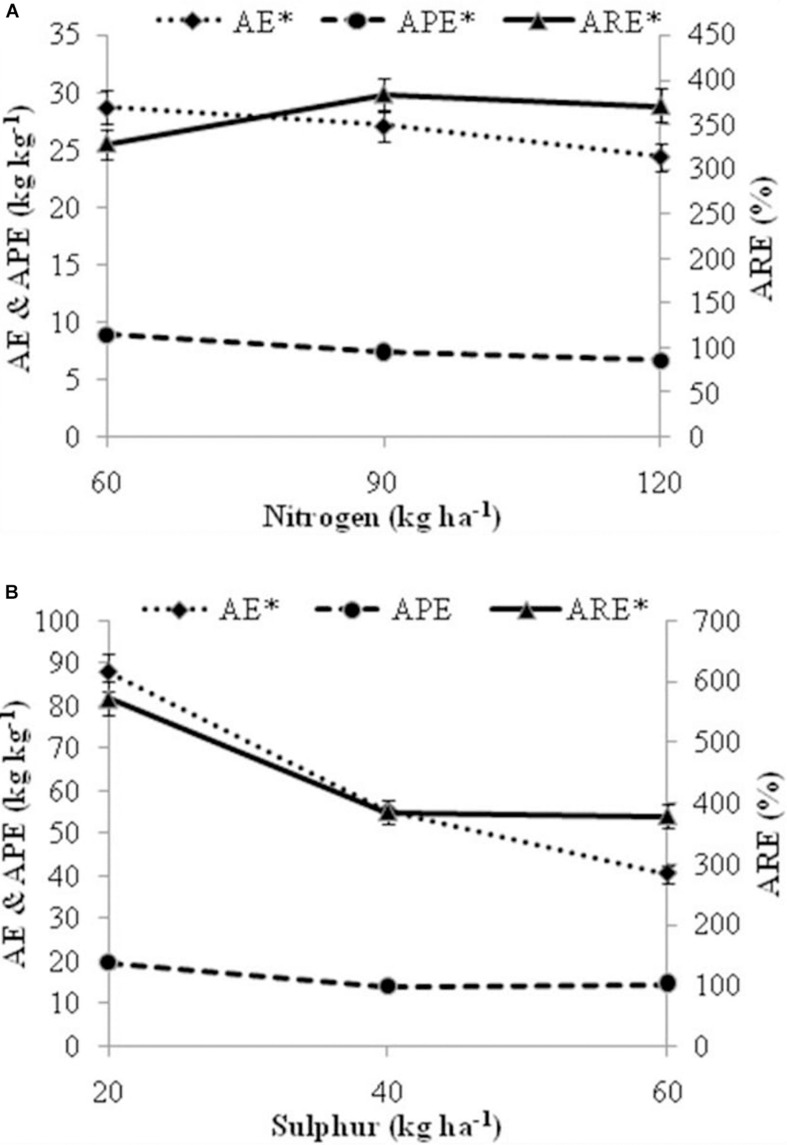
Effect of fertilization levels **(A)** levels of nitrogen **(B)** levels of sulfur on nutrient efficiency traits *viz.* AE, Agronomic efficiency; APE, Agro physiological efficiency; ARE, Apparent recovery efficiency. The mean values of 2 years pooled data are presented. Asterisk (*) on nutrient efficiency traits indicates significant differences among fertilizer levels at *P* = 0.05. Vertical bars indicate a mean standard error (±).

The effect of nutrient application on nutrient use efficiency (NUE) traits was found to be significant; except for agro physiological efficiency (APE) in case of S levels (Figure 8). Agronomic efficiency (AE) of nutrients reduced with higher N and S doses. Among N levels, significantly higher AE (28.70 kg kg^–1^) and APE (8.95 kg kg^–1^) was found with N at 60 kg ha^–1^ than other doses. Apparent recovery efficiency (ARE) values for N levels varied from 328.05 to 383.31%, and was found significantly higher in N at 90 kg ha^–1^ which further decreases with increase in N level. Among S levels, significantly higher AE (87.76 kg kg^–1^) and ARE (571.71%) was recorded at 20 kg ha^–1^ S than other doses.

## Discussion

### Growth and Yield Parameters

Growth parameters were higher in first year during all the observations (Table 2), due to favorable environmental conditions like temperature and humidity for plant growth during 2018. In this study, relative humidity was recorded higher in first cropping year (Figure 1), which might have reduced stomata function and subsequently reduces the growth attributes. These results are in line with Mortensen and Fjeld (1995). Irrespective of significantly higher growth parameters in first cropping year, yield parameters were significantly higher in second cropping year during harvest (Table 3). This is because in our study rainfall was higher throughout the first cropping year, i.e., from mid of July to September as compared to second cropping season (Figure 1), which might have deteriorated the plants in excessive water and lead to reduced biomass and oil yield.

Among the nutrient applications, N and S at higher dose recorded significantly higher growth and yield parameters (Tables 2, 3). The promotional influence of N on growth parameters is due to enhanced growth of meristematic cells leading to development of shoots besides plant height. Omidbaigi et al. (2008) and Singhal and Sharma (2010) observed positive effect of N fertilization on vegetative parameters of wild marigold, reporting higher and heavier plants in N treated plots as compared to control. In our results higher doses of N and S recorded higher yield. This is because in our results higher growth parameters were recorded in higher dose of N which has lead to higher vegetative growth, which resulted in increased herbage production and EO content, consequently, EO yield increased to greater extent. These results were in line with Chrysargyris et al. (2016) in lavender (*Lavandula angustifolia* Mill.). Due to better availability of S at higher doses, enhance activities of different enzyme occur which lead to synthesis of more protein, increasing growth parameters. Kumar et al. (2010) while working on *M. arvensis* recorded higher plants with more branches and leaf area index due to higher S application. Increase in S doses had major impact on biomass and oil yield of sweet basil *O. basilicum* with highest yield beyond 80 kg ha^–1^ S in Oliveira et al. (2014).

Essential oil content was significantly higher in second cropping season (Table 3). This is because of slight drop in temperature during second year during reproductive phase which controls the synthesis of EO, as environment plays a major role in it. At flowering stage low temperature during night reduces the EO synthesis (Najem et al., 2011). N at 120 kg ha^–1^ recorded higher EO content; these results can be because the application of N is directly involved in the vegetative growth of plant which results in increasing the proportion of leafy parts in the herb which will lead to more leaf to stem ratio as compared to control plants. Since in *Tagetes* EO is mostly present in leaves and flowers, so plants with N application and more leaf+flower to stem ratio will have increased EO content as compared to control plants. These results were in line with Kucukyumuk et al. (2015) in lavandin (*Lavandula intermedia* L.). Application of higher dose of S recorded higher EO content because S has major function in synthesis of proteins which further led enhanced EO content (Oliveira et al., 2014).

### Stomatal and Leaf Oil Glands Characteristics

Stomatal density was significantly higher in control while stomatal length was higher in plots with nutrient application (Table 4). It occurred because higher N and S lead to higher growth with bigger cells, which lead to increase in size of stomata. On the same view range, SEM brought about lower stomatal density and lower stomatal length using higher dosage of nutrient than the control. Numbers of oil glands were higher in S treated plots than control, while N did not show any significant effect (Table 4). This is because S application increases oil and protein contents in plant by higher availability of other nutrients as P, K, Zn which has major role in the growth of reproductive organs and regulation of oil glands (Malhi et al., 2007).

### Essential Oil Composition

Our results showed that *Z-β-*ocimene, dihydrotagetone, tagetone, and ocimenone were the major chemical components in *T. minuta* EO similar to the previous researcher’s studies (Pazcel et al., 2018; Rathore et al., 2018). In this experiment, percentage of major components of *Tagetes*, EO was altered by the fertilizer application but in terms of quality at 120 kg N ha^–1^ and 60 kg S ha^–1^ were best. The relative percentage of major compound *Z-β-*ocimene (35–50%) is important in determining the quality of *T. minuta* EO at the international market (Cornelius and Wycliffe, 2016). Significant differences were observed in EO composition of *T. minuta* with different N and S doses. Our results reported that major compound *Z-β-*ocimene increases with higher N dose and found maximum in N at 120 kg ha^–1^ (Table 6). N fertilization may have enhanced the EO biosynthesis process through its direct or indirect role in plant metabolism which resulted in more plant metabolites. Nutrients like N and S promote terpenoid emissions by promoting electron transport rate and leaf photosynthesis which provide ATP requirements and carbon substrate availability for isoprene synthesis. All carbon-based secondary metabolites ultimately depend on CO_2_ fixation and, as a result, a relationship between nutrients and stored terpenoids (Ormeno and Fernandez, 2012). According to carbon nutrient balance hypothesis (CNBH) carbon and nutrient availability in the plant environment determines the production of metabolites. Limited nutrient resources curtailed plant growth, rather than photosynthesis, resulting in an excess of carbohydrates. Under such conditions, the CNBH asserts that the excess of carbohydrates is not used for growth but provides, instead, an additional substrate to synthesize defense secondary metabolites (Bryant et al., 1983). However, as per growth differentiation balance hypothesis (GDBH) under soils rich in nutrient resources, growth (biomass production), will be favored over differentiation (cell maturation and production of defensive compounds). As nitrogen becomes scarcer, differentiation will predominate, and consequently terpenoid accumulation will increase at the expense of growth, since the plant allocates proportionately more of an abundant resource, such as carbon, to the acquisition of the scarce resource or secondary metabolites, resulting in more terpenoid synthesis (Lorio, 1986). Similar to our results major constituent dihydrotagetone decreases with higher N dose above 50 kg ha^–1^, with increase in limonene, (Z)-tagetone and (Z)-tagetenone (Singh et al., 2008). Omidbaigi et al. (2008) observed that EO from 100 kg N ha^–1^ contained highest amount of dihydrotagetone (57.1%). Significantly higher percentage of *Z*-β-ocimene was reported with 60 kg S ha^–1^ (Table 6). S application increases oil and protein contents in plant with higher growth of reproductive organs and regulation of oil glands which may have led to variation among EO components. The major chemical components of EO of *O. basilicum* changed as the S increased, the concentration of linalool and eugenol increased in comparison with the control (Oliveira et al., 2014). Kumar et al. (2010) while studying *M. arvensis* reported 70% higher menthol in NPK+Zn+S than NPK+Zn, showing direct influence of S on major component.

### Plant Nutrient Concentration, Available Nutrients in Soil and Nutrient Use Efficiency Traits

The concentration of nutrients in plant parts steadily increases with rise in N and S doses (Figure 7). Better supply of N and S facilitates root growth by synthesis of auxin and cytokinin (Krouk et al., 2010), leading to elimination of huge nutrients from small and deep area of soil. Higher biomass yield with moderate concentration of nutrients in plant parts can also be the source of higher absorption of nutrients under rising N and S dosage. Application of N recorded statistical results on available N, P, and S in soil (Table 7). The higher the value for soil N supply the more likely it is that the microorganisms in a soil will convert more organic N into mineral N for plant uptake. N decomposition, provide acidic compounds, with more available nutrients in the soil (Rakshit et al., 2015). These results were in line with Kamble and Kathmale (2015) in onion. Similarly, in our results S application recorded significant effect on available N and S, while no effect was seen on available P and K (Table 7). This is because deficiency in S decreases the N efficiency; and higher S dose will increase the use efficiency of nitrogenous fertilizer, increasing availability of N and S in soil. Fazili et al. (2008) and Baligar et al. (2001) also reported higher nutrient availability in soil due to application of N and S fertilizers in oilseed crops similar to our results. The NUE traits decline with raising nutrient levels (Figure 8). A declining pattern in AE was observed with raising N from 30 to 120 kg ha^–1^ (Arduini et al., 2006) and 120 to 360 kg ha^–1^ (Belete et al., 2018). Zemichael et al. (2017) also reported decreasing trend in NUE traits with increasing doses of nutrients, explaining better performance with idea of supplying N at maximum absorption period resulting in higher biomass production while, supplying N at higher doses at planting time results in reduced uptake of N and S loses of nutrient with reduced efficiency.

## Conclusion

The results reveal that N and S application alter the biomass yield and essential oil yield, and secondary metabolite profile of *T. minuta* under western Himalayas. Over highest N and S fertilizer application rate (i.e., 120 kg N ha^–1^ and 60 kg S ha^–1^) total biomass yield was 14.48 and 6.74% higher than that under no fertilizer application (N0 and S0), respectively. Essential oil gland density showed marked improvement with the application of S upto 40 kg ha^–1^. On the other hand, application of 120 kg N ha^–1^ and 40 kg S ha^–1^ registered about 50.08 and 18.27% higher essential oil yield, respectively, compared with control. These application increases the availability and uptake of nutrients with low NUE traits over those achieved under control in the present study. Substantial variations in major compounds (*Z*-β-ocimene and dihydrotagetone) of essential oil were also observed in this experiment. Thus, it can be concluded that higher N (120 kg ha^–1^) and intermediate S (40 kg ha^–1^) may be adopted to enhance the biomass and essential oil yield of *T. minuta* with desired quality. However, further studies are required to understand the unknown pathways of these nutrients in aromatic crops in enhancing physiological (oil gland characteristics), yield and quality characteristics.

## Data Availability Statement

The original contributions presented in the study are included in the article/supplementary material, further inquiries can be directed to the corresponding author.

## Author Contributions

SW: experiment execution, data collection, data processing, oil analysis, identification of compounds, soil analysis, statistical analysis, and literature search and manuscript writing. RK: develop the idea, designing the experiment, overall supervision of the experiment, data processing, and manuscript editing. Both authors contributed to the article and approved the submitted version.

## Conflict of Interest

The authors declare that the research was conducted in the absence of any commercial or financial relationships that could be construed as a potential conflict of interest.
